# Comparative Study of Damage Detection Methods Based on Long-Gauge FBG for Highway Bridges

**DOI:** 10.3390/s20133623

**Published:** 2020-06-28

**Authors:** Shi-Zhi Chen, De-Cheng Feng, Wan-Shui Han

**Affiliations:** 1 Highway College, Chang’an University, Xi’an 710064, China; hws@gl.chd.edu.cn; 2 Key Laboratory of Concrete and Prestressed Concrete Structures of the Ministry of Education, Southeast University, Nanjing 210096, China; dcfeng@seu.edu.cn

**Keywords:** long-gauge FBG, damage detection, highway bridges, vehicle–bridge interaction, comparative study

## Abstract

Damage detection of highway bridges is a significant part of structural heath monitoring. Conventional accelerometers or strain gauges utilized for damage detection have many shortcomings, especially their monitoring gauge length being too short, which would result in poor damage detection results. Under this circumstance, long-gauge FBG sensors as a novel optical sensor were developed to measure the macro-strain response of the structure. Based on this sensor, many derived damage detection methods were proposed. These methods exhibit various characteristics and have not been systematically compared. As a result, it is difficult to evaluate the state of the art and also leads to confusion for users to select. Therefore, a strict comparative study on three representative methods using long-gauge FBG was carried out. First, these methods’ theoretical backgrounds and formats were reformulated and unified for better comparison. Then, based on validated vehicle–bridge coupling simulation, these methods’ performances were tested through a series of parametric studies including various damage scenarios, vehicle types, speeds, road roughness and noise levels. The precision and reliability of three methods have been thoroughly studied and compared.

## 1. Introduction

Nowadays, in-service highway bridges always suffer from damage caused by external effects like normal traffic and material degradation. Without appropriate maintenance actions, this damage would inevitably result in severe disaster, causing human and fortune losses. In order to detect potential damage and avoid these losses, the conception of structural health monitoring (SHM) was proposed. By installing sensors on a highway bridge, the SHM technique could identify potential damage and assess its condition through monitored structural response [1].

Damage detection methods, as a core part of SHM, have drawn a lot of attention. Currently, there have been plenty of methods proposed utilizing accelerometers or strain gauges [2]. For the methods based on accelerometers, the core part is extracting modal information of a highway bridge like intrinsic frequencies and modal shapes from acceleration response through modal identification algorithms, such as frequency domain decomposition (FDD), stochastic subspace identification (SSI) [3,4,5]. Owing to the intrinsic relationship between modal information and structural physical parameters, these methods could achieve the goal of damage detection. Salawu [6] and Doebling et al. [7] have systematically summarized this kind of method.

After being verified through numerical simulations and indoor experiments, these methods have also been tested in actual bridges. However, they were found to be insensitive to structural local damage. Huth et al. [8] conducted a modal test on a severely damaged prestressed concrete bridge. Its modal shapes were found to be nearly unchanged compared with those when the bridge is intact. Through analyzing modal shape and other derivative parameters, such as modal curvature and modal strain energy, Alvandi and Cremona [9] found out that modal information would be sensitive to local damage only if there is no interference of noise. When introducing noise in monitored response, the modal information would no longer reflect the structural condition clearly. Chang et al. [10] carried out a destruction test on an actual bridge and discovered that in order to succeed in detecting damage, the order of modal information should be high enough. However, with current test technique, it is difficult to accurately obtain high order modal information. Due to these reasons, the performance of accelerometer based methods is not satisfying in practical application.

In contrast with modal information, strain response is directly related to structural local conditions. Hence, inherently, the methods based on strain gauge would be more sensitive to structural local damage [11]. Based on strain response, Cardini and Dewolf [12] deduced the neutral axis height of bridges in order to detect bridge damage. Catbas et al. [13] also proposed a damage detection method utilizing the correlations between strain time histories and verified this method in a bridge test. Li’s [14] review has thoroughly collected this kind of method. Although strain gauge-based methods are more sensitive to local damage, in practice these methods have a common weakness that conventional strain gauge’s monitoring range is too short. As a result, it is hard to cover all potential damaged area on a bridge with finite number of strain gauges. In the meantime, more sensors would be demanded to increase the possibility of damage identification, but corresponding problems of installation inconvenience would emerge [15]. Conventional strain gauge also has other shortcomings, like being fragile and sensitive to electromagnetic interference (EMI) [15,16].

To solve these problems, distributed optical fiber (DOF) sensors such as Brillouin optical time domain reflectometer (BOTDR) were used for bridge damage detection [17]. Normally they can achieve long-distance distributed sensing with a precision of 5 με, which is very suitable for large-scale structures like bridges. However, their sampling frequencies are rather low, less than 1 Hz, which is not capable of bridge dynamic monitoring. Moreover, the spatial resolution of these techniques is larger than 500 mm, making it quite discrepant to ideal distributed sensing [18]. Therefore, the advantage of these sensors would be diminished. In comparison with these distributed optical fiber sensors, the fiber Bragg grating (FBG) sensor as another kind of fiber optic sensor can realize high sampling frequency, fulfilling the demand of bridge SHM. Currently, the main drawback of FBG is its short monitoring gauge, which would lead to same problems met in the conventional strain gauge [19]. Moreover, the fiber-based sensors, especially FBG, are usually fragile, which is not durable under actual environment. These drawbacks restrained their application.

In order to expand monitoring gauge and increase durability of FBG, Li and Wu [20] designed a special packaging structure and invented the long-gauge FBG sensor. It is the only sensor which can be used to monitor the average strain response within its monitoring gauge, which is defined as macro-strain. Long-gauge FBG sensor’s monitoring gauge length could vary from 100 mm to 1000 mm according to relevant monitoring demands. Meanwhile, due to the spatial resolution existing in DOF sensors, the performance of long-gauge FBG is quite similar with normal DOF sensors, so it could be treated as a quasi-DOF sensor. Owing to these advantages, long-gauge FBG has been gradually applied in bridge SHM [21,22,23]. Based on long-gauge FBG, some exclusive damage detection methods have also been proposed, which can be found in many studies [24,25]. These methods were developed separately and exhibited various characteristics. Although they were alleged to be feasible and reliable, there is not a comparative study among them, especially regarding their precision and reliability. Consequently, it is difficult to evaluate the state of the art, resulting in some confusion for users to select.

Therefore, this paper conducts a strict comparison on three representative damage detection methods based on long-gauge FBG. The methods employed in this study were proposed by Wu et al. [26], Hong et al. [27] and Chen et al. [28]. For better comparison, these methods’ formats were firstly reformulated and unified. Then, a numerical simulation based on vehicle–bridge interaction was programmed and validated by an indoor experiment in order to carry out a series of parametric studies. The parameters considered include various damage scenarios, vehicle types, speeds, road surface roughness degrees and noise levels. The performances of different methods under various scenarios are tested and compared.

The paper is organized as follows: Section 2 introduces the unified theoretical background for each method. Section 3 presents the vehicle–bridge interaction theory used for simulation. To rectify the reliability of the simulation, an indoor experimental validation was also illustrated in Section 3. The numerical simulation and results discussion are given in Section 4 and Section 5 draws the final conclusions.

## 2. Theoretical Background

### 2.1. Long-Gauge FBG

For better demonstration of each damage detection method based on long-gauge FBG, the basic structure and characteristics of long-gauge FBG are presented first.

**Figure 1:**
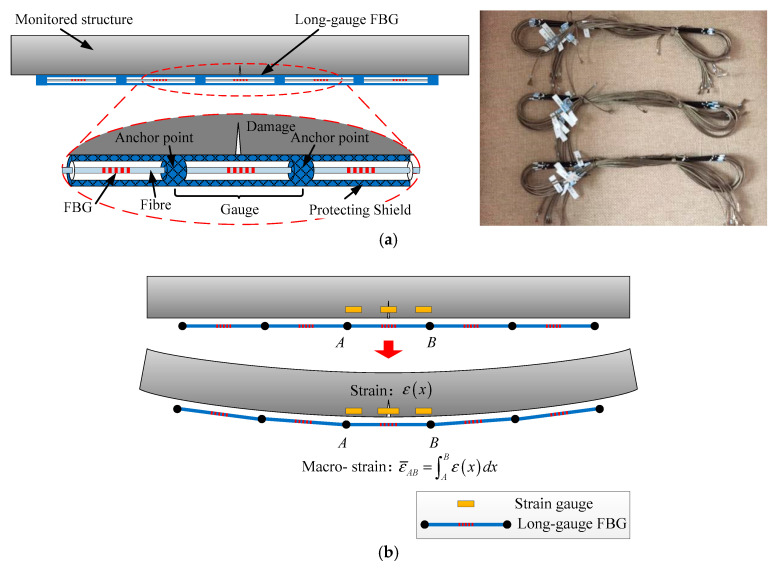
Schematic diagram of long-gauge fiber Bragg grating (FBG) sensor: (**a**) Basic structure and actual view of long-gauge FBG; (**b**) Comparison between long-gauge FBG and strain gauge.

The basic structure of long-gauge FBG is displayed in Figure 1a. It can be seen that the FBG as core sensing unit of long-gauge FBG is protected and isolated by a shield tube made by basalt fibers from the external environment. Hence, the durability of fragile FBG can be effectively enhanced. Meanwhile, the FBG is attached to the monitored structure only at two anchor points. At two anchor points, the inner fiber was solidified to the basalt fiber tube by epoxy resin. Owing to this special design, the long-gauge FBG can measure the average strain within the area between two anchor points. This area is defined as its monitoring gauge. In actual practice, in order to monitor large scale structures like bridges, long-gauge FBG sensors can also be multiplexed, forming a sensor sequence, as shown in Figure 1b. Compared with conventional strain gauge, one main merit of long-gauge FBG is that, by multiplexing, they can cover the whole span of a bridge while conventional strain gauge can only monitor some certain section of a bridge. As can be seen in Figure 1b, when the damage area was not covered by a strain gauge, the damage could not be detected. However, the long-gauge FBG can always successfully monitor the potential damage, even though damage would randomly occur.

**Figure 2:**
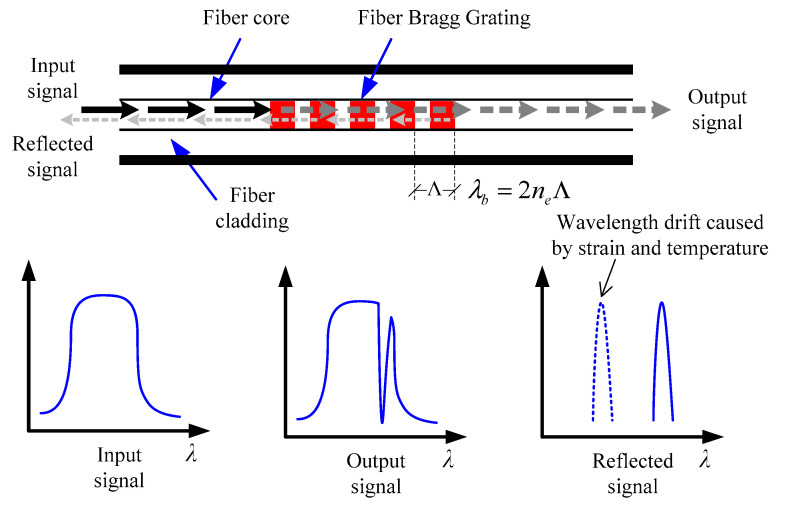
Optical characteristic of long-gauge FBG.

As for its optical characteristics, they can be seen in Figure 2. The FBG within the gauge functions like a narrow-band reflective mirror. When a beam of light was inputted into long-gauge FBG, only the light with its wavelength equal to FBG’s central wavelength *λ_b_* would be reflected. The central wavelength *λ_b_* is determined by fiber core’s effective refractive index *n_e_* and grating period Λ, as shown in Equation (1).

(1) $$\lambda_{b}=2n_{e}\Lambda$$

When the long-gauge FBG deforms with monitored structure, the grating period Λ would change accordingly, so as the central wavelength *λ_b_*. The relationship between the change of central wavelength and deformation is:

(2) $$\left[\frac{\Delta \lambda_{b}}{\lambda_{b}}\right]=C_{S}\cdot \Delta \varepsilon +C_{T}\cdot \Delta T$$

where Δ*λ_b_* is the change of central wavelength, Δ*ε* is the strain monitored, Δ*T* is the change of temperature, *C_S_* and *C_T_* are strain factor and temperature factor, respectively, which can be calibrated by experiment.

According to the structure of long-gauge FBG, the relationship between measured structural average strain ${\bar{\varepsilon }}_{AB}$ and strain response at each point can be deduced, as shown in Figure 1b.

(3) $${\bar{\varepsilon }}_{AB}=\frac{1}{l_{AB}}\int_{A}^{B}\varepsilon \left(x\right)\;dx$$

in which *ε*(*x*) represents the strain of point *x*, *l_AB_* represents the distance between *A* and *B*. The average strain ${\bar{\varepsilon }}_{AB}$ is also defined as macro-strain. Currently, long-gauge FBG is the only way to measure the macro-strain response of the structure.

In Figure 1b, the main advantage of long-gauge FBG has also been explained. In order to capture the random damage feature of the structure, large amounts of conventional strain gauges are demanded, while long-gauge FBG can achieve covering large structural area with a finite number of sensors.

### 2.2. Macro-Strain Influence Line

**Figure 3:**
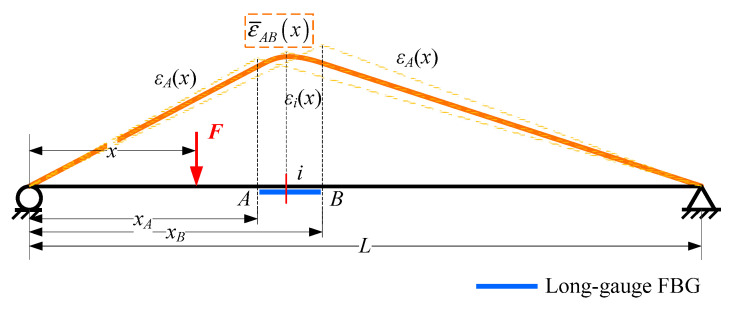
Strain and macro-strain influence line response.

After long-gauge FBG was developed, many derived damage detection methods have been carried out. Because moving vehicles are the main source of load on a bridge, all typical methods discussed in this study utilized the macro-strain influence line as their theoretical foundations. According to structural mechanics, the strain influence line response *ε_i_* (*x*) generated by a moving unit concentration force *F* at a certain section *i* of a simply supported beam (Figure 3) would be:

(4) $$\varepsilon_{i}\left(x\right)=\{\begin{array}{cc} \frac{\left(L-x_{i}\right)h}{{\left(EI\right)}_{i}L}\cdot x & 0<x\leq x_{i}\\ \frac{\left(L-x\right)h}{{\left(EI\right)}_{i}L}\cdot x_{i} & x_{i}<x\leq L \end{array}$$

in which *L* is the span length of a beam, *x_i_* is the coordinate of section *i*, *h* is the height of neutral axis and (*EI*)*_i_* is the bending stiffness of section *I*.

Based on Equation (3), strain influence line response can be modified to macro strain influence line response ${\bar{\varepsilon }}_{AB}\left(x\right)$:

(5) $${\bar{\varepsilon }}_{AB}\left(x\right)=\{\begin{array}{cc} \frac{\left(x_{B}-x_{A}\right)\left(2L-x_{B}-x_{A}\right)h}{2{\left(\bar{EI}\right)}_{AB}Ll_{g}}\cdot x & 0<x\leq x_{A}\\ \frac{h}{2{\left(\bar{EI}\right)}_{AB}Ll_{g}}\cdot \left[-Lx^{2}+\left(2Lx_{B}+x_{A}^{2}-x_{B}^{2}\right)x-Lx_{A}^{2}\right] & x_{A}<x\leq x_{B}\\ \frac{\left(x_{B}^{2}-x_{A}^{2}\right)h}{2{\left(\bar{EI}\right)}_{AB}Ll_{g}}\cdot \left(L-x\right) & x_{B}<x\leq L \end{array}$$

where *x_A_* and *x_B_* are two end coordinates of a long-gauge FBG, *l_g_* is the gauge length of long-gauge FBG sensor and ${\left(\bar{EI}\right)}_{AB}$ represents the average bending stiffness within monitoring gauge *AB*.

**Figure 4:**
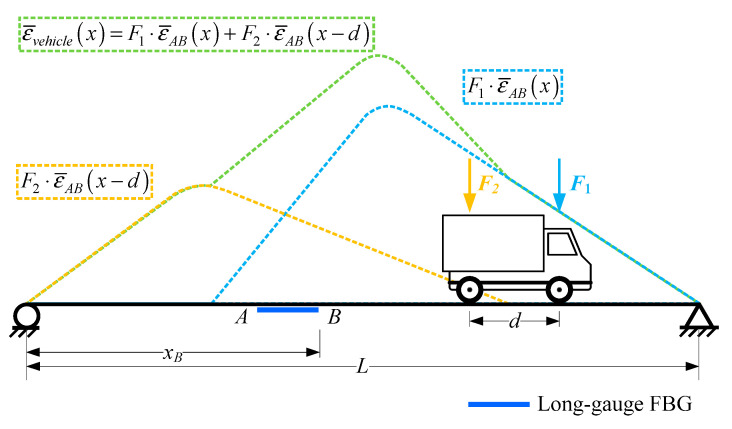
Macro-strain response caused by a vehicle.

For a vehicle passing through a bridge, its response would be the superposition of its axial weights multiplying macro-strain influence line response, as illustrated in Figure 4, because the bridge would be still in the elastic stage when a vehicle passes through. The macro-strain response caused by a vehicle could be expressed as:

(6) $$\begin{array}{ccc} {\bar{\varepsilon }}_{vehicle}\left(x\right) & = & F_{1}\cdot {\bar{\varepsilon }}_{AB}\left(x\right)+F_{2}\cdot {\bar{\varepsilon }}_{AB}\left(x-d_{1}\right)+\cdots +F_{n}\cdot {\bar{\varepsilon }}_{AB}\left(x-\sum_{k=1}^{n-1}d_{k}\right)\\ & = & \sum_{i=1}^{n}F_{i}\cdot {\bar{\varepsilon }}_{AB}\left(x-\sum_{k=1}^{i-1}d_{k}\right) \end{array}$$

where *F_i_* is the *i*th axial weight, *n* is the total axle number of a vehicle, *d_k_* is the *k*th wheelbase of a vehicle.

**Figure 5:**
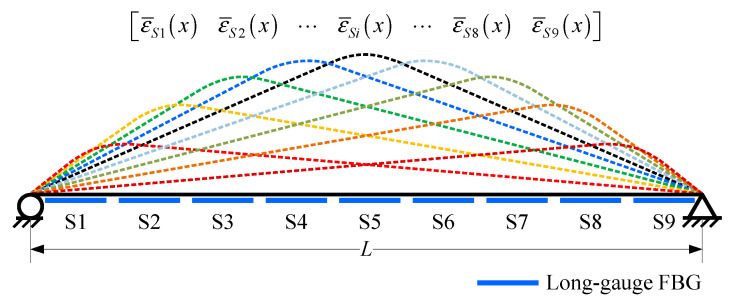
Macro-strain influence line responses obtained by long-gauge FBG sequence.

Because the macro-strain response caused by a vehicle is just the superposition of a single influence line, they would exhibit the same characteristic. Hence, the damage detection method can be established by studying the basic features of single macro-strain influence line response. Then, with a long-gauge FBG sequence attached on a beam, a series of macro-strain responses would be obtained, which can be used for developing damage detection methods, as shown in Figure 5. Then, the damage detection methods discussed in this study were proposed.

### 2.3. Damage Detection Method (M1)

The damage index proposed in Wu et al. [26] was called macro-strain influence line response envelope (MIE). It consists of the maximum of each macro-strain response obtained by a long-gauge FBG sensor: $\left[\begin{array}{ccccc} \max\left({\bar{\varepsilon }}_{S1}\right) & \cdots & \max\left({\bar{\varepsilon }}_{Si}\right) & \cdots & \max\left({\bar{\varepsilon }}_{S9}\right) \end{array}\right]$. Based on Equation (5), as the geometric parameters such as *L*, *l_g_*, *x_A_* and *x_B_* are fixed, the maximum of macro-strain response is only inversely proportional to the average bending stiffness ${\left(\bar{EI}\right)}_{AB}$ within each monitoring gauge, because when the sensor was installed these parameters would be invariant. Therefore, the maximum of macro-strain response can be expressed as:

(7) $$\max({\bar{\varepsilon }}_{Si})=\frac{F\left(x_{A},x_{B},L,l_{g},h\right)}{{\left(\bar{EI}\right)}_{Si}}$$

in which *F*(*x_A_*, *x_B_*, *L*, *l_g_*, *h*) is an implicit function of geometric parameters.

**Figure 6:**
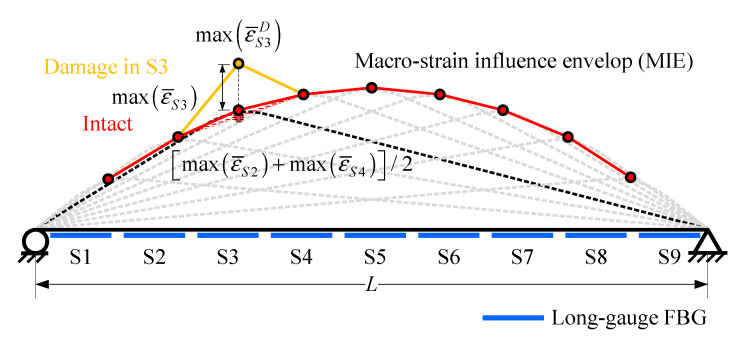
MIE obtained from long-gauge FBG sequence.

If there is damage causing *β* degree of stiffness degradation, the corresponding MIE would emerge as a peak at the relevant sensor, as displayed in Figure 6, because after damage the maximum of macro-strain response would be:

(8) $$\max\left({\bar{\varepsilon }}_{Si}^{D}\right)=\frac{F\left(x_{A},x_{B},L,l_{g},h\right)}{{\left(\bar{EI}\right)}_{Si}^{D}}=\frac{F\left(x_{A},x_{B},L,l_{g},h\right)}{\left(1-\beta \right){\left(\bar{EI}\right)}_{Si}}=\frac{1}{\left(1-\beta \right)}\cdot \max\left({\bar{\varepsilon }}_{Si}\right)$$

where superscript *D* represents the parameter under the damaged scenario.

Due to this mechanism, the damage location can be detected, as shown in Figure 6. The damage degree can be calculated through:

(9) $$\beta =1-\frac{\max\left({\bar{\varepsilon }}_{Si}\right)}{\max\left({\bar{\varepsilon }}_{Si}^{D}\right)}$$

This is the core part of the damage detection method proposed by Wu et al. [26]. This method uses the MIE of the macro-strain response caused by a vehicle to locate and quantify the damage. However, in order to accurately quantify the damage extent, the MIE under intact conditions is needed for reference, which is often hard to get for an actual bridge. Therefore, Wu et al. [26] selected the average value of two nearby intact areas’ MIE to approximate the intact MIE of the identified damage area:

(10) $$\max\left({\bar{\varepsilon }}_{Si}^{I}\right)\approx \left[\max\left({\bar{\varepsilon }}_{Si-1}\right)+\max\left({\bar{\varepsilon }}_{Si+1}\right)\right]/2$$

### 2.4. Damage Detection Method (M2)

Instead of MIE, the damage detection method proposed by Hong et al. [27] selected integrals of the macro-strain influence line response (IMIL) as the damage index. Through derivation based on Equation (5), IMIL would be:

(11) $$\begin{array}{cc} \int_{0}^{L}{\bar{\varepsilon }}_{AB}\left(x\right)\;dx & =\int_{0}^{x_{A}}\frac{\left(x_{B}-x_{A}\right)\left(2L-x_{B}-x_{A}\right)h}{2{\left(\bar{EI}\right)}_{AB}Ll_{g}}\cdot x\;dx+\int_{x_{A}}^{x_{B}}\frac{h}{2{\left(\bar{EI}\right)}_{AB}Ll_{g}}\cdot \left[-Lx^{2}+\left(2Lx_{B}+x_{A}^{2}-x_{B}^{2}\right)x-Lx_{A}^{2}\right]\;dx\\ & +\int_{x_{B}}^{L}\frac{\left(x_{B}^{2}-x_{A}^{2}\right)h}{2{\left(\bar{EI}\right)}_{AB}Ll_{g}}\cdot \left(L-x\right)\;dx\\ & =-\frac{h}{12{\left(\bar{EI}\right)}_{AB}Ll_{g}}\cdot \left(x_{A}-x_{B}\right)\cdot \left\{\begin{array}{l} 3x_{a}{}^{3}+\left(3x_{b}-8L-6\right)\cdot x_{a}{}^{2}+\left[3L^{2}+\left(12-2x_{b}\right)\cdot L-6x_{b}\right]\cdot x_{a}\\ +L\cdot x_{b}\cdot \left(3L-2x_{b}\right) \end{array}\right\}\\ & =\frac{G\left(x_{A},x_{B},L,l_{g},h\right)}{{\left(\bar{EI}\right)}_{AB}} \end{array}$$

Similarly, IMIL is also inversely proportional to the average bending stiffness ${\left(\bar{EI}\right)}_{AB}$. After the bending stiffness within *i*th sensor decreases, the corresponding IMIL would be:

(12) $$IMIL_{Si}^{D}=\frac{G\left(x_{A},x_{B},L,l_{g},h\right)}{{\left(\bar{EI}\right)}_{Si}^{D}}=\frac{G\left(x_{A},x_{B},L,l_{g},h\right)}{\left(1-\beta \right){\left(\bar{EI}\right)}_{Si}}=\frac{1}{\left(1-\beta \right)}\cdot IMIL_{Si}$$

**Figure 7:**
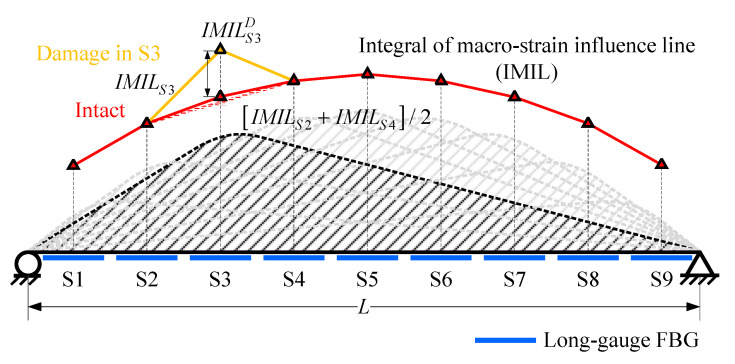
Influence line response (IMIL) obtained from long-gauge FBG sequence.

Equation (12) means if there is a damage causing *β* degree of stiffness degradation, the corresponding IMIL would fluctuate, as shown in Figure 7. This is the basic theory of the damage detection methods proposed by Hong et al. [27]. The damage extent can be calculated through:

(13) $$\beta =1-\frac{IMIL_{Si}}{IMIL_{Si}^{D}}$$

Likewise, Hong et al. [27] selected the average of two nearby intact areas’ IMIL to substitute the intact IMIL reference needed for damage extent calculation.

(14) $$\max\left({\bar{\varepsilon }}_{Si}^{I}\right)\approx \left[\max\left({\bar{\varepsilon }}_{Si-1}\right)+\max\left({\bar{\varepsilon }}_{Si+1}\right)\right]/2$$

### 2.5. Damage Detection Method (M3)

Through analyzing the feature of macro-strain influence line response (Equation (5)), Chen et al. [28] realized that Equation (5) is a second order function of *x* only when *x* is between *x_A_* and *x_B_*. As a result, the second order difference of macro-strain influence line response (SODM) is derived as damage index:

(15) $$\frac{d^{2}{\bar{\varepsilon }}_{AB}\left(x\right)}{dx^{2}}=\{\begin{array}{cc} 0 & 0<x\leq x_{A}\\ -\frac{h}{{\left(\bar{EI}\right)}_{AB}l_{g}} & x_{A}<x\leq x_{B}\\ 0 & x_{B}<x\leq L \end{array}$$

**Figure 8:**
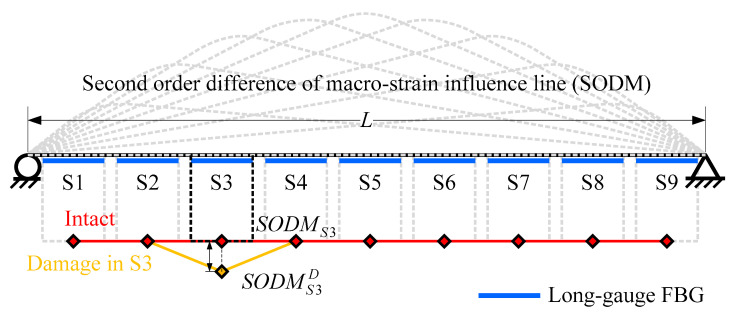
SODM obtained from long-gauge FBG sequence.

Compared with the original macro-strain influence line response and two derivatives used in former methods, the expression of SODM is much more concise. The relationship between SODM and average bending stiffness is quite clear. The SODM obtained from long-gauge FBG sequence is illustrated in Figure 8. The peak of SODM would reflect the location of damage. The extent of damage would be:

(16) $$\beta =1-\frac{SODM_{Si}}{SODM_{Si}^{D}}$$

Because SODM forms a straight line, unlike a curve formed by MIE and IMIL, the method proposed by Chen et al. [28] can directly calculate the damage extent through Equation (16) without any approximate assumption.

According to the introductions mentioned above, it can be discovered that, theoretically, all these three methods would detect damage location and extent based on monitored macro-strain response through long-gauge FBG. All their theoretical foundations come from macro-strain influence line response. One evident difference among them is that methods proposed by Wu et al. [26] and Hong et al. [27] need to adopt an approximation for damage extent estimation because intact condition reference of the structure is hard to get, while the method proposed by Chen et al. [28] has no need. Except this, the difference among their characteristics cannot be revealed merely based on their theoretical backgrounds. In order to better compare these methods, a refined vehicle–bridge coupling simulation is needed to generate macro-strain response caused by moving vehicles under various parametric scenarios. The basic theory of vehicle–bridge coupling simulation would be introduced first in the next section.

## 3. Vehicle–Bridge Coupling Simulation

### 3.1. Vehicle–Bridge Coupling Simulation Theory

**Figure 9:**
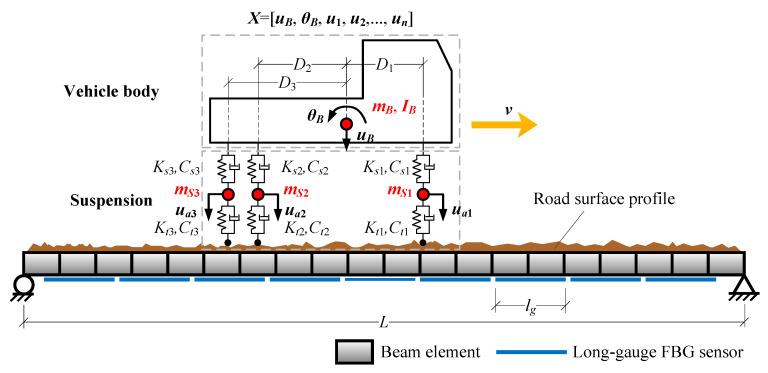
Vehicle–bridge coupling simulation.

Compared with treating a vehicle as a group of moving loads, in vehicle–bridge coupling simulation, a vehicle is represented by a multi-degree of freedom model (Figure 9) containing the displacement of the vehicle body and suspension structures. The stiffness and damping of suspension structure and tire can also be effectively reflected. Besides these, the vehicle–bridge coupling simulation could reasonably consider the interaction effect between a vehicle and a bridge. Therefore, the bridge response simulated considering vehicle–bridge coupling would be more suitable for comparing the performance of these methods.

Figure 9 demonstrates a schematic diagram of a vehicle–bridge coupling simulation. A two dimensional three-axle vehicle model moves at a velocity ***v*** over a bridge covered by long-gauge FBG sensors. According to D’Alembert’s principle, the equation of motion of a vehicle would be:

(17) $$\left[\begin{array}{cc} M_{V1} & 0\\ 0 & M_{V2} \end{array}\right]\cdot \ddot{X}+\left[\begin{array}{cc} C_{V11} & C_{V12}\\ C_{V21} & C_{V22} \end{array}\right]\cdot \dot{X}+\left[\begin{array}{cc} K_{V11} & K_{V12}\\ K_{V21} & K_{V22} \end{array}\right]\cdot X=-\left[\begin{array}{c} 0\\ F\left(t\right) \end{array}\right]+\left[\begin{array}{c} 0\\ F_{G} \end{array}\right]$$

in which $X={\left[u_{B},\theta_{B},u_{a1},u_{a2},\cdots ,u_{an}\right]}^{T}$ is the response vector of a vehicle. **F**(*t*) is the interaction force between vehicle axle and bridge. **F***_G_* is vehicle’s static axial weight. **M***_V_*, **C***_V_*, **K***_V_* are sub-matrices of vehicular mass, damping and stiffness matrices, respectively.

For a bridge, it can be simplified as an Euler-Bernoulli beam to simulate its mechanical behavior. A bridge’s equation of motion would be:

(18) $$M_{B}\cdot \ddot{D}+C_{B}\cdot \dot{D}+K_{B}\cdot D=I\cdot F$$

where **M***_B_*, **C***_B_* and **K***_B_* are mass, damping and stiffness matrices of the bridge, respectively. **D** is the displacement vector, and **I** is an interpolating matrix, which would equivalently allocate the axial force of a vehicle to relevant nodes of a bridge.

According to the equilibrium of the interaction force between a bridge and a vehicle, a vehicle’s equation of motion (Equation (17)) and a bridge’s equation of motion (Equation (18)) can be coupled together forming a vehicle–bridge coupling system’s equation of motion:

(19) $$M^{C}\left(t\right)\cdot \ddot{U}+C^{C}\left(t\right)\cdot \dot{U}+K^{C}\left(t\right)\cdot U=F\left(t\right)$$

where **U** = {**X**^T^ **D**^T^}^T^ is the coupled displacement vector. **M***^C^*(*t*), **C***^C^*(*t*) and **K***^C^*(*t*) are mass, damping and stiffness matrices of vehicle–bridge coupling system.

Utilizing Newmark-*β* method, Equation (19) can be solved to obtain the dynamic response ***D***(*t*) of a bridge under a moving vehicle. Then, based on the plane section assumption, the dynamic strain response *ε*(*x*,*t*) at point *x* under the bridge can be derived from ***D***(*t*):

(20) $$\varepsilon \left(x,t\right)=h\cdot \frac{\partial^{2}D\left(x,t\right)}{\partial x^{2}}$$

where *ε*(*x*,*t*) and *D*(*x*,*t*) are the strain and displacement response of a bridge at point *x*, respectively. *h* is the neutral axis height.

In order to simulate the macro-response caused by a long-gauge FBG sensor, simulated strain response *ε*(*x*,*t*) needs to be expanded into macro-strain response ${\bar{\varepsilon }}_{AB}$. According to the definition of macro-strain in Equation (3), the macro-strain response caused by a vehicle can be deduced:

(21) $$\begin{array}{ccc} {\bar{\varepsilon }}_{AB} & = & \frac{1}{l_{AB}}\int_{A}^{B}\varepsilon \left(x,t\right)\;dx\\ & = & \frac{1}{l_{AB}}\int_{A}^{B}h\cdot \frac{\partial^{2}D\left(x,t\right)}{\partial x^{2}}\;dx\\ & = & \frac{\bar{h}}{l_{AB}}I\left(x\right)\cdot D\left(t\right) \end{array}$$

in which $\bar{h}$ is the average neutral axis height and $I\left(x\right)={\left[\begin{array}{ccccccccc} 0 & \cdots & -1 & 0 & \cdots & 1 & 0 & \cdots & 0 \end{array}\right]}_{1\times n}$ is an interpolation vector.

Moreover, road surface roughness is an important factor, which would dramatically affect the bridge response caused by a moving vehicle (Figure 9). In order to consider this factor, a displacement power spectral density (PSD) defined by relevant specification is utilized to generate road surface roughness by inverse fast Fourier transformation [29]. By selecting different spectral roughness coefficient values, various classes of road surface roughness can be simulated. In this study, 5 classes A, B, C, D, E are selected representing very good, good, average, poor and very poor road surface roughness conditions, as shown in Table 1.

Then, according to basic theory of vehicle–bridge coupling simulation, the macro-strain response of a bridge measured by long-gauge FBG under the effect of a moving vehicle can be programmed and simulated in MATLAB. More specific explanations of the vectors and matrixes mentioned in this section can be found in Chen et al. [23]. In order to validate the correctness of the simulated macro-strain response, an indoor vehicle–bridge coupling experiment was carried out, which will be introduced in the next part.

### 3.2. Experimetental Validation

**Figure 10:**
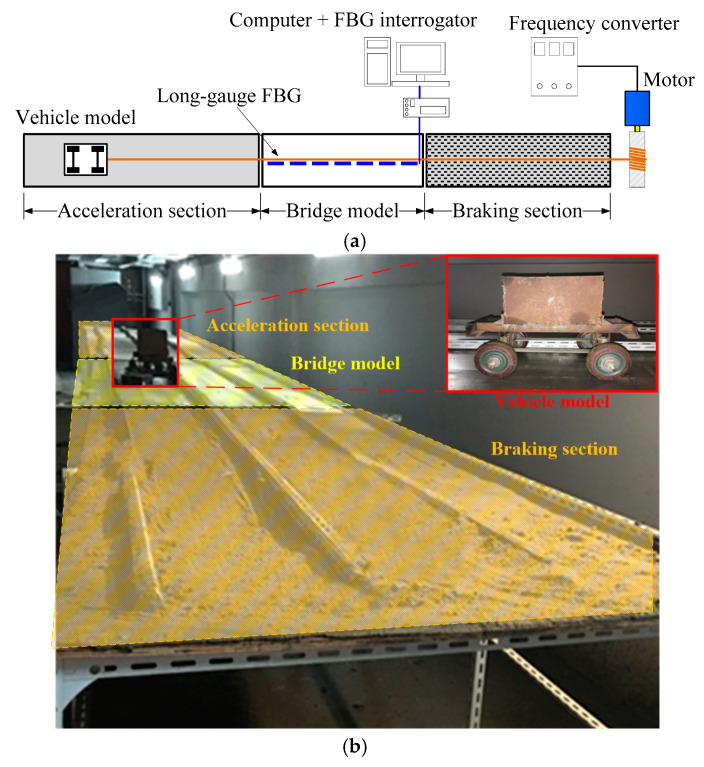
Vehicle–bridge coupling experimental platform: (**a**) Schematic diagram; (**b**) Actual view.

In order to validate the correctness of numerical results generated by vehicle–bridge coupling simulation, an indoor experiment was conducted. The whole experimental platform is displayed in Figure 10. The platform has three sections: the acceleration section, bridge model and braking section. The bridge model is fabricated by polymethyl-methacrylate and its span length is 3 m. It is simply supported by two supports. A total of 9 long-gauge FBG sensors with 300 mm gauge length were installed underneath the bridge model to capture the macro-strain response of the bridge model.

A vehicle model is dragged by a motor and moves through a bridge model. The macro-strain response generated by the moving vehicle model is collected through a FBG interrogator fabricated by Micro Optics Inc., whose type is SM530 (Micro-optics, Inc., Hackettstown NJ, USA). In experiments, the velocity of the vehicle model can be precisely controlled by changing the rotation frequency of the motor using a frequency converter.

**Figure 11:**
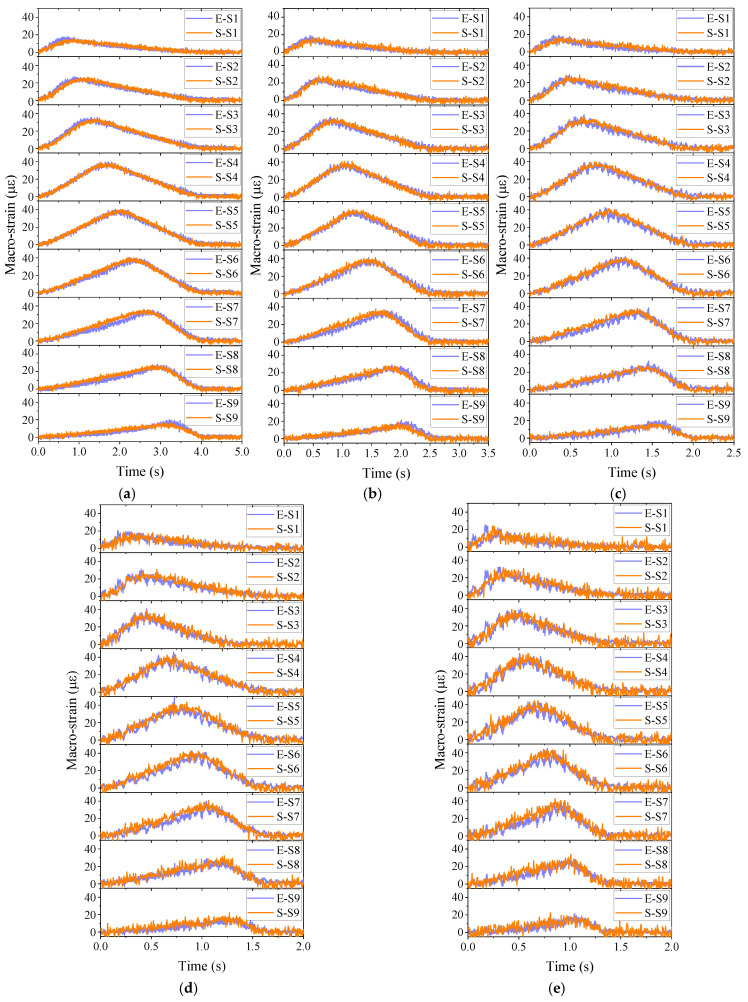
Comparison between typical experimental and simulated macro-strain results: vehicle velocity: (**a**) 10 km/h; (**b**) 15 km/h; (**c**) 20 km/h; (**d**) 25 km/h; (**e**) 30 km/h.

In the experiment, macro-strain responses under different vehicle moving scenarios were collected by long-gauge FBG. They were utilized to validate the vehicle–bridge coupling simulation programmed. The comparison between typical experimental and simulation results is presented in Figure 11. In the legend of Figure 11, “E-S*i*” and “S-S*i*” represent the corresponding experimental and simulated macro-strain responses of sensor S*i*, respectively. It can be found that the macro-strain response simulated agrees well with the response captured in the experiment. Under various vehicle velocities, the difference between experimental and simulated results varied little, which implies that the vehicle–bridge coupling simulation is suitable for conducting comparative studies of damage detection methods. Therefore, in next section, a series of numerical simulations were introduced to compare the performance of three damage detection methods introduced in Section 2 based on the model parameters validated through this indoor experiment.

## 4. Numerical Simulation

### 4.1. Simulation Scenario

**Figure 12:**
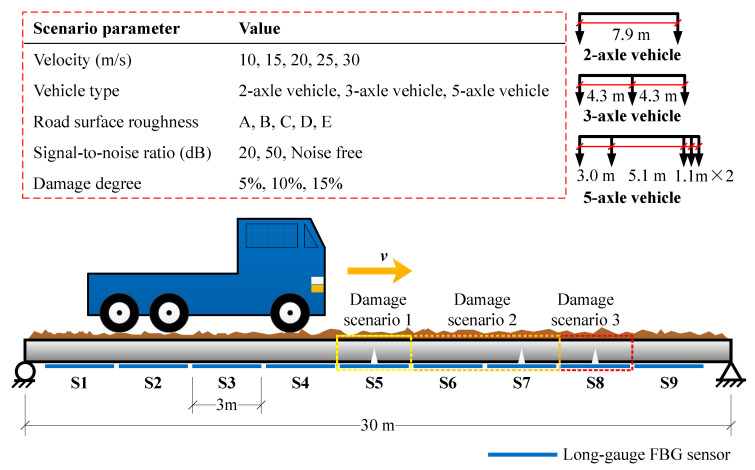
Damage scenarios designed in numerical simulation.

For better comparing the performances of three damage detection methods mentioned in Section 2, a series numerical simulations were carried out with the model parameters validated by an indoor experiment. The corresponding model parameters validated from experiment are listed in Table 2. These parameters also referred to relevant studies [30,31,32]. Three damage scenarios with various impact factors are designed in this simulation, as shown in Figure 12, containing single damage and multiple damage conditions. In addition, three typical damage extents: 5%, 10% and 15%, are considered. Moreover, the potential impact factors for damage detection are taken into account, such as vehicle type, speed, road surface roughness and signal-to-noise ratio.

**Figure 13:**
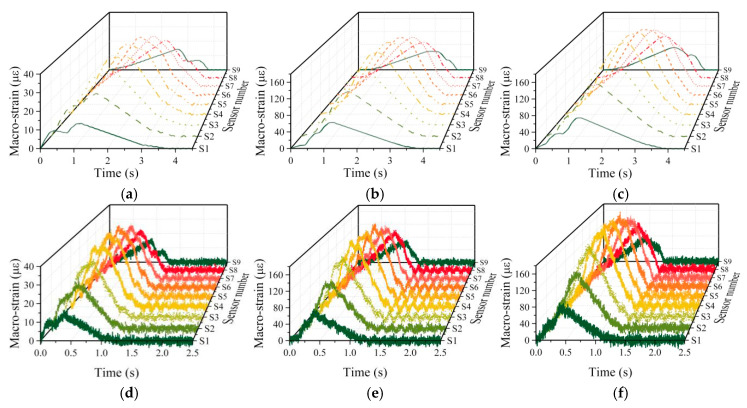
Typical macro-strain response obtained through vehicle-coupling simulation: Noise-free signal of (**a**) 2-axle vehicle; (**b**) 3-axle vehicle; (**c**) 5-axle vehicle with 10 m/s on A road surface; 20 signal-to-noise ratio signal of (**d**) 2-axle vehicle; (**e**) 3-axle vehicle; (**f**) 5-axle vehicle with 30 m/s on E road surface.

The typical macro-strain responses obtained are shown in Figure 13. It can be seen that macro-strain response varies dramatically under various parametric scenarios, which would be very suitable for testing the method’s performance. Their performance would be impacted more or less. Then, inputting these data into three damage detection methods, corresponding results would be obtained for comparison. The discussion of results is given in the next section.

### 4.2. Results Discussion

#### 4.2.1. Comparison under Different Vehicle Types

**Figure 14:**
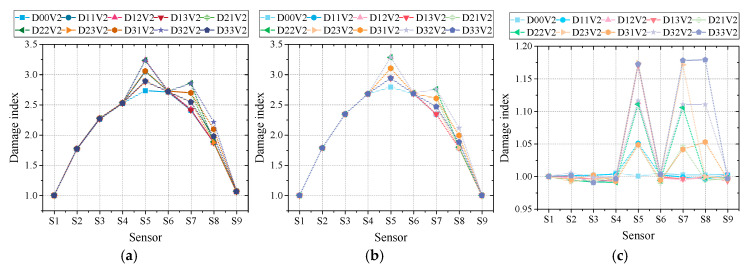

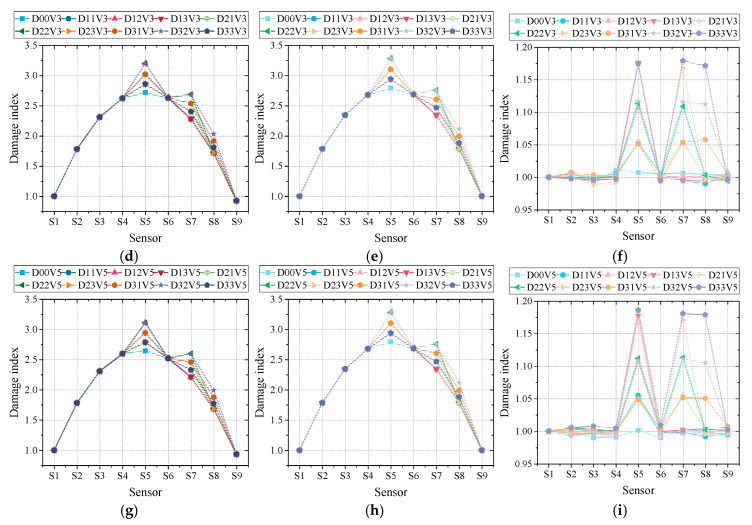
Damage detection results: (**a**) M1 with 2-axle vehicle; (**b**) M2 with 2-axle vehicle; (**c**) M3 with 2-axle vehicle; (**d**) M1 with 3-axle vehicle; (**e**) M2 with 3-axle vehicle; (**f**) M3 with 3-axle vehicle; (**g**) M1 with 5-axle vehicle; (**h**) M2 with 5-axle vehicle; (**i**) M3 with 5-axle vehicle.

Firstly, in the numerical simulation, three different typical vehicle types are considered to investigate its influence. For brevity, the methods proposed by Wu et al. [26], Hong et al. [27] and Chen et al. [28] are represented by M1, M2 and M3. The corresponding damage indexes of three methods MIE, IMIL and SODM under three vehicle types are displayed in Figure 14. They were calculated based on Equations (8), and (12) and (15). The letters ‘D’ and ‘V’, with numbers behind in legends, represent the damage scenario and vehicle type. The other parameters like speed, road surface roughness, noise level in Figure 14 are set as 10 m/s and A class without noise.

From Figure 14, it can be seen that the overall performance of three methods under different vehicle types is rather good. The damage location in each damage scenario can be reflected by the corresponding damage index. However, it can be discovered that the damage index of M1 showed some fluctuations under three vehicle types. Moreover, the shape of M1’s damage index is not ideally symmetric as introduced in its basic theory (Figure 6), while M2’s damage index remains more stable and symmetric. In addition, compared with two curved lines of damage indexes obtained in M1 and M2, M3’s damage index is more convenient for calculating damage extent. The damage index of M3 still has some tiny fluctuations, which would influence the accuracy of damage extent calculation. Then, using Equations (9), (13) and (16), the damage extent measured through the three methods can be calculated, which are listed in Table 3. The value within brackets is the relative error.

According to Table 3, it can be found that the performance of M1 and M2 is very poor with the lowest relative error larger than 15%. The main reason is when the intact condition of the bridge is unknown, an approximation is adopted that uses the average of nearby intact gauges’ indexes approximate intact condition, as given in Equations (10) and (14). This assumption itself would introduce huge error, because the damage index curve of M1 and M2 is a quadratic curve rather than a straight line. In contrast, M3’s performance is rather good owing to its not needing an intact condition reference. As for their performance under different vehicle types, M2’s results are the most stable, which seems to be immune to the change of vehicle type. Meanwhile, M1 and M3’s results change under different vehicle types. Between these two methods, M1’s performance is steadier, though its accuracy is much poorer than that of M3. In general, the performance with 2-axle vehicles is a little bit weaker than those with the other two vehicle types. This might be the result of the response signal being more easily polluted if its amplitude is smaller. In this study, the weight of two-axle vehicle is the lightest, leading to the smallest response amplitude.

In addition, their performance under different damage scenarios is also given in Table 3. Under nine designed damage scenarios, all three methods exhibit a similar trend in that, with the damage extent rising, the detection results become more accurate. Meanwhile, the relative error in sensor S5 is the lowest, exceeding 100% under different damage scenarios. This might be the result of the response amplitude in S5 being higher than those in S7 and S8. Besides this trend, it can be seen that M1 and M2’s relative error in S8 is far beyond those in S5 and S7. The reason is that in damage scenarios concerned with sensor S8, the nearby sensor S7 also suffered from damage. Under this situation, when calculating the damage extent of S8, the damage index of S6 is selected as S6 is the nearest intact gauge. Consequently, more approximation error is introduced into the results of S8.

Moreover, once the intact condition is known for reference, the damage extent of M1 and M2 would become much more ideal as listed in Table 4. Under this circumstance, the accuracy of M1 and M2 is better than that of M3. M2 exhibits the most precise and stable performance. Nevertheless, it still needs to be clarified that the excellent performance of these two methods is based on the precondition that bridge’s intact condition is known beforehand.

Overall, under different damage scenarios and vehicle types, all three methods can successfully detect the damage location. However, for damage extent calculation, unless intact condition reference is available, the performance of M1 and M2 is unacceptable. As for the impact of vehicle type, M3’s performance is a little bit weak, while M2’s is the most stable. In addition, the impact of damage location on three damage detection methods is more obvious than that of vehicle type. The damage detection results in S7 and S8 are worse than that in S5 because the corresponding response amplitude is smaller. Due to the same reason, the relative errors in 15% damage scenarios are the lowest.

#### 4.2.2. Comparison under Different Speeds and Road Surface Roughness

**Figure 15:**
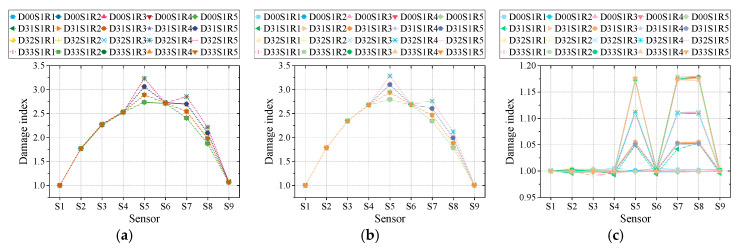

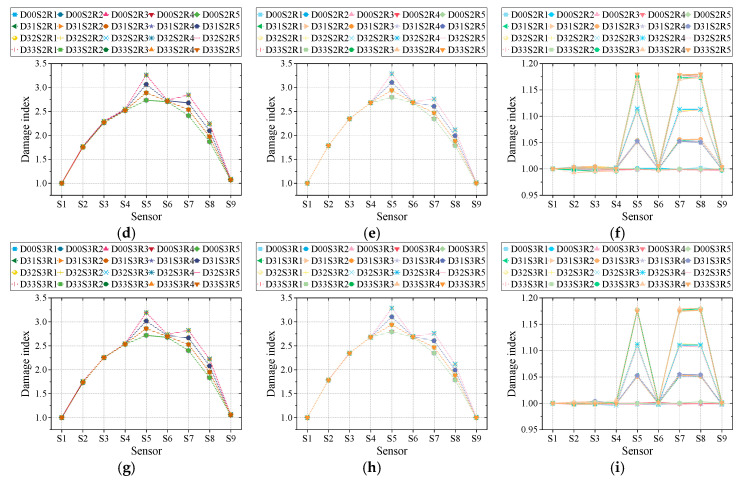
Damage detection results: (**a**) M1 with 2-axle vehicle; (**b**) M2 with 2-axle vehicle; (**c**) M3 with 2-axle vehicle; (**d**) M1 with 3-axle vehicle; (**e**) M2 with 3-axle vehicle; (**f**) M3 with 3-axle vehicle; (**g**) M1 with 5-axle vehicle; (**h**) M2 with 5-axle vehicle; (**i**) M3 with 5-axle vehicle.

Based on the findings in last section, the parameter vehicle type and damage scenario is fixed as two-axle vehicle and scenario 3 to test the performance of three methods under various speeds and road surface roughness. The reason why these two factors are considered together is that there might be some couple effect within these two factors impacting the performance of damage detection methods. Corresponding damage indexes of three methods under various speeds and road surface roughness are displayed in Figure 15. The letters ‘S’ and ‘R’, with numbers behind in legends, represent speed and roughness degree. The noise level is set as noise-free.

According to the results presented in Figure 15, it can be revealed that vehicle speed and road surface roughness’ influence on the performance of three methods is limited. Moreover, the coupling effect of these two factors was not discovered. These findings proved these methods’ reliability in actual stochastic traffic flow to some degree, which accords with the findings in previous works [24,25,26,27,28]. Among three methods, M2 behaves the best, whose results remained stable under various speeds and road surface roughness. Similar to the findings in the last section, M3’s behavior is less ideal with some fluctuations in results. Similarly, through Equations (7), (11) and (14), the damage extent can be calculated, as listed in Table 5. It needs to be clarified that in Table 5 the results of M1 and M2 are based on intact condition reference, because the results without intact condition proved to be incomparable in the last section.

According to Table 5, all three methods’ performance under various speeds and road surface roughness conditions are good. The accuracy of M2 is the best. Similar to the findings in the last section, the performance of M3 is relatively unstable. The detection error varies under different scenarios. This demonstrates that, as for stability, M3’s performance is not ideal, while M1 and M2’s excellent performance is based on the precondition that intact condition was known in advance. Therefore, although M3’s performance is not very stable, its not needing an intact condition reference still made it an alternative method.

In summary, all three methods showed good performance under different vehicle speeds and road surface roughness, except that M3’s behavior exhibited little fluctuations.

#### 4.2.3. Comparison under Different Signal-to-Noise Ratios

**Figure 16:**
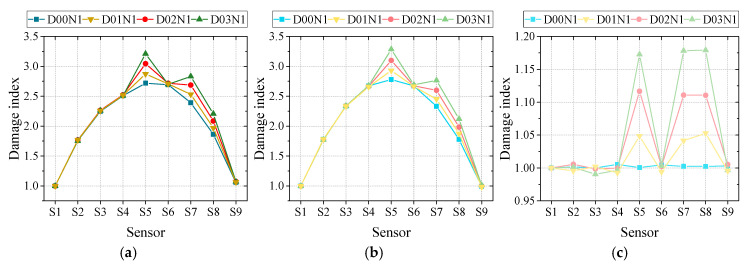

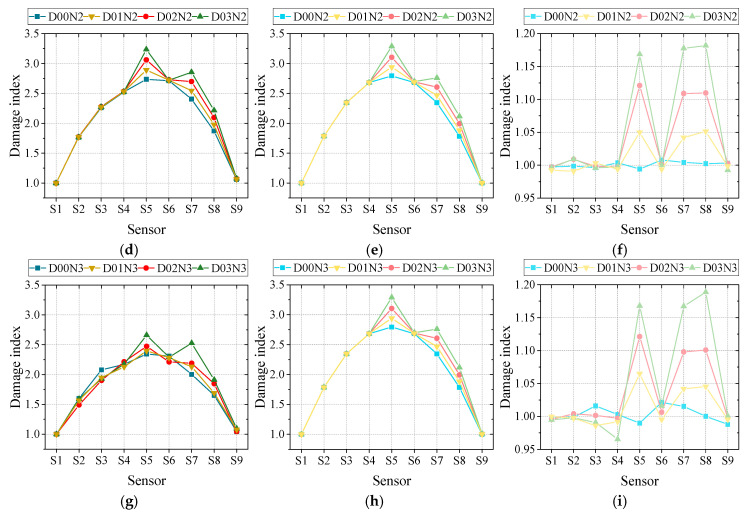
Damage detection results: (**a**) M1 noise-free; (**b**) M2 noise-free; (**c**) M3 noise-free; (**d**) M1 with 50 signal-to-noise ratio; (**e**) M2 with 50 signal-to-noise ratio; (**f**) M3 with 50 signal-to-noise ratio; (**g**) M1 with 20 signal-to-noise ratio; (**h**) M2 with 20 signal-to-noise ratio; (**i**) M3 with 20 signal-to-noise ratio.

At last, the influence of noise level on the three methods was studied in order to illustrate the three methods’ performance under adverse environments. The noise level was controlled by signal-to-noise level, which was set as 20, 50 and noise-free to mimic various noise conditions, as designed in previous studies. According to the definition of signal-to-noise level, with the value decreasing, the noise level would rise accordingly. For better comparison, at this part the other factors such as vehicle type, speed and road surface roughness are set as two-axle vehicle with 10 m/s under A class road surface roughness. Corresponding damage indexes of three methods under various noise levels are displayed in Figure 16.

As can be seen in Figure 16, the influence of noise level on the three damage detection methods varied dramatically. M2 is the most robust one facing different noise levels, while M1 and M3’s performance is less ideal. Especially in noise level 3, with signal-to-noise level reaching 20, evident abnormal fluctuations were observed in M1 and M3’s damage detection results. Then, utilizing Equations (7), (11) and (14), the damage extent can be obtained as given in Table 6. It also needs to be elucidated that the results of M1 and M2 in Table 6 are on the basis of intact condition reference due to the same reason explained in Section 4.2.2.

According to Table 6, the findings in Figure 16 are certified. M2’s are the most stable and accurate under three noise levels with the largest error less than 3%. Meanwhile, the performance of M1 and M3 degrades intensely as noise level rises. From noise-free to 20 signal-to-noise ratio, the relative error of M1 is raised from 3.6% to 56.40% for the 5% damage extent scenario. For the more severe 15% damage extent scenario, the relative error dropped to around 20%, which is a little bit lower but still unacceptable. For M3, the overall performance is better than M1, while its relative error still reached 40% for the 5% damage extent scenario with a 20 signal-to-noise ratio. Similarly, the accuracy under more severe damage scenarios is much better for M3. In summary, M2’s robustness under different noise levels is the best among the three methods. M3 and M1’s performance under different noise levels is very unstable, while M1 is the worst one.

## 5. Conclusions

Long-gauge FBG, as a novel optical sensor with many advantages, has been applied in SHM. Many exclusive damage detection methods have been proposed utilizing this sensor and exhibited various characteristics. Although they were alleged to be feasible and reliable, there is not a comparative study among them, especially regarding their precision and reliability. As a result, it is difficult to evaluate the state of the art, which also results in some confusion for users to select. Therefore, a strict comparison of three typical damage detection methods based on long-gauge FBG was conducted. A numerical simulation based on vehicle–bridge interaction was programmed and validated by an indoor experiment. The performances of different methods under various scenarios were tested and compared through a series of parametric numerical studies. The following conclusions can be drawn:

(1) The damage detection methods proposed by Wu et al. [26] (M1), Hong et al. [27] (M2) and Chen et al. [28] (M3) as three representative methods were selected to conduct this comparative study. Firstly, these three methods’ theoretical backgrounds were reformulated and unified for comparison. It can be discovered that, theoretically, all these three methods would detect damage location and extent based on monitored macro-strain response. All their theoretical foundations come from macro-strain influence line, while the damage indexes they selected are derivatives of the macro-strain influence line. One evident difference among them is that M1 and M2 need to adopt a approximation for damage extent estimation because the intact condition of the structure is hard to get, while M3 has no need.

(2) In order to compare the performances of the three methods under various critical parameters, a parametric study based on vehicle–bridge interaction was programmed and validated. The parameters considered in this study include damage scenario, vehicle type, speed, road surface roughness and noise level. Under different damage scenarios and vehicle types, all of them can successfully detect the damage location. However, for damage extent calculation, unless intact condition reference is available, the accuracy of M1 and M2 is very poor. As for the impact of vehicle type, although M3’s precision is a little bit weak, it is still the most stable one. In addition, the impact of damage location on three damage detection methods is more obvious than that of vehicle type.

(3) As for the influence of vehicle speed and road surface roughness, all three methods showed good performance under different vehicle speeds and road surface roughness, except that M3’s behavior exhibited little fluctuations. At last, as for the impact of noise level, M2’s robustness under different noise levels is the best among the three methods. M3 and M1’s performance under different noise levels is very unstable, while M1 is the worst one. All in all, the M2’s performance is the most eminent only if the intact condition reference is available. Meanwhile M3 is a little bit unstable but it has a merit that the intact condition reference is not needed.

## Figures and Tables

**Table 1:** Different classes of road surface roughness.

Class	Spectral Roughness Coefficient	Road Surface Roughness Sample
A	0 m^3^/cycles	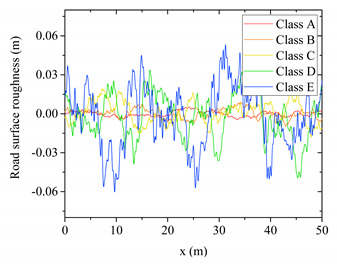
B	1 × 10^−6^ m^3^/cycles	
C	6 × 10^−6^ m^3^/cycles	
D	16 × 10^−6^ m^3^/cycles	
E	64 × 10^−6^ m^3^/cycles	

**Table 2:** Parameters of vehicle–bridge coupling simulation.

Bridge Model		Vehicle Model			
*L*	30 m	*m*_B_	260 kg	*I*_B_	500 kg·m^2^
*E*	3.023 × 10^4^ MPa	*D*_1_	1.8 m	*D*_2_	1.8 m
*I*	5.697 × 10^11^ mm^4^	*m*_S1_	10 kg	*m*_S2_	10 kg
*A*	1.138 × 10^6^ mm^2^	*k*_S1_	4.15 × 10^3^ N/m	*k*_S2_	4.15 × 10^3^ N/m
*h*	2000 mm	*k*_T1_	3.89 × 10^3^ N/m	*K*_T2_	3.89 × 10^3^ N/m
ρ	1.171 × 10^4^ g/mm^3^	*C*_S1_	39.5 N/m/s	*C*_S2_	39.5 N/m/s
ξ	0.02	*C*_D1_	42.0 N/m/s	*C*_D2_	42.0 N/m/s

**Table 3:** Damage extent detection results of three methods with different vehicles.

Designed Damage Extent (%)		Detected Damage Extent (%)								
		M1			M2			M3		
		V2	V3	V5	V2	V3	V5	V2	V3	V5
5	S5	9.11 (82.20%)	8.16 (63.20%)	8.10 (62.00%)	8.73 (74.60%)	8.73 (74.60%)	8.73 (74.60%)	4.65 (7.00%)	5.20 (4.00%)	4.76 (4.80%)
	S7	9.58 (91.60%)	9.45 (89.00%)	9.68 (93.60%)	9.37 (87.40%)	9.37 (87.40%)	9.37 (87.40%)	4.39 (12.20%)	5.28 (5.60%)	5.27 (5.40%)
	S8	36.23 (624.60%)	34.56 (591.20%)	34.99 (599.80%)	34.69 (593.80%)	34.69 (593.80%)	34.69 (593.80%)	5.02 (0.40%)	5.49 (9.80%)	4.82 (3.60%)
10	S5	13.91 (39.10%)	12.93 (29.30%)	12.89 (28.90%)	13.45 (34.50%)	13.45 (34.50%)	13.45 (34.50%)	10.24 (2.40%)	9.66 (3.40%)	9.68 (3.20%)
	S7	14.44 (44.40%)	14.17 (41.70%)	14.30 (43.00%)	13.96 (39.60%)	13.96 (39.60%)	13.96 (39.60%)	9.58 (4.20%)	9.84 (1.60%)	10.15 (1.50%)
	S8	39.56 (295.60%)	37.99 (279.90%)	38.50 (285.00%)	38.15 (281.50%)	38.15 (281.50%)	38.15 (281.50%)	9.96 (0.40%)	10.12 (1.20%)	9.52 (4.80%)
15	S5	18.68 (24.53%)	17.77 (18.47%)	17.68 (17.87%)	18.16 (21.07%)	18.16 (21.07%)	18.16 (21.07%)	14.56 (2.93%)	14.96 (0.27%)	15.10 (0.67%)
	S7	19.27 (28.47%)	18.86 (25.73%)	18.88 (25.87%)	18.54 (23.60%)	18.54 (23.60%)	18.54 (23.60%)	14.65 (2.33%)	14.37 (4.20%)	14.60 (2.67%)
	S8	42.90 (186.00%)	41.49 (176.60%)	42.07 (180.47%)	41.65 (177.67%)	41.65 (177.67%)	41.65 (177.67%)	15.20 (1.33%)	14.65 (2.33%)	15.19 (1.27%)

**Table 4:** Damage extent detection results of M1 and M2 with intact reference.

Designed Damage Extent (%)		Detected Damage Extent (%)					
		M1			M2		
		V2	V3	V5	V2	V3	V5
5	S5	5.18 (3.60%)	4.97 (0.60%)	5.12 (2.40%)	5.00 (0.00%)	5.00 (0.00%)	5.00 (0.00%)
	S7	5.42 (8.40%)	5.17 (3.40%)	5.17 (3.40%)	5.00 (0.00%)	5.00 (0.00%)	5.00 (0.00%)
	S8	5.42 (8.40%)	5.22 (4.40%)	5.20 (4.00%)	5.21 (4.20%)	5.21 (4.20%)	5.20 (4.20%)
10	S5	10.38 (3.80%)	10.00 (0.00%)	10.13 (1.30%)	10.00 (0.00%)	10.00 (0.00%)	10.00 (0.00%)
	S7	10.79 (7.90%)	10.44 (4.40%)	10.13 (1.30%)	10.00 (0.00%)	10.00 (0.00%)	10.00 (0.00%)
	S8	10.68 (6.80%)	10.37 (3.70%)	10.40 (4.00%)	10.44 (4.40%)	10.44 (4.40%)	10.44 (4.40%)
15	S5	15.54 (3.60%)	15.07 (0.47%)	15.08 (0.53%)	15.00 (0.00%)	15.00 (0.00%)	15.00 (0.00%)
	S7	15.78 (5.20%)	15.29 (1.93%)	15.02 (0.13%)	15.00 (0.00%)	15.00 (0.00%)	15.00 (0.00%)
	S8	15.52 (3.47%)	15.53 (3.53%)	15.62 (4.13%)	15.71 (4.73%)	15.71 (4.73%)	15.70 (4.67%)

**Table 5:** Damage extent detection results of three methods under various speed and roughness.

Designed Damage Extent (%)		Detected Damage Extent (%)								
		M1			M2			M3		
		S1	S2	S3	S1	S2	S3	S1	S2	S3
5	R1	5.18 (3.60%)	5.32 (6.40%)	4.37 (12.60%)	5.00 (0.00%)	5.01 (0.20%)	5.00 (0.00%)	5.02 (0.40%)	4.76 (4.80%)	4.62 (7.60%)
	R2	5.18 (3.60%)	5.32 (6.40%)	4.37 (12.60%)	5.00 (0.00%)	5.01 (0.20%)	5.00 (0.00%)	5.06 (1.20%)	4.81 (3.80%)	5.10 (2.00%)
	R3	5.18 (3.60%)	5.32 (6.40%)	4.37 (12.60%)	5.00 (0.00%)	5.01 (0.20%)	5.00 (0.00%)	5.11 (2.20%)	4.98 (0.40%)	5.01 (0.20%)
	R4	5.18 (3.60%)	5.32 (6.40%)	4.37 (12.60%)	5.00 (0.00%)	5.01 (0.20%)	5.00 (0.00%)	4.91 (1.80%)	4.79 (4.20%)	4.86 (2.80%)
	R5	5.18 (3.60%)	5.32 (6.40%)	4.37 (12.60%)	5.00 (0.00%)	5.01 (0.20%)	5.00 (0.00%)	5.01 (0.20%)	5.13 (2.60%)	4.62 (7.60%)
10	R1	10.38 (3.80%)	10.73 (7.30%)	9.35 (6.50%)	10.00 (0.00%)	10.01 (0.10%)	10.00 (0.00%)	10.24 (2.40%)	10.44 (4.40%)	9.90 (1.00%)
	R2	10.38 (3.80%)	10.73 (7.30%)	9.35 (6.50%)	10.00 (0.00%)	10.01 (0.10%)	10.00 (0.00%)	9.98 (0.20%)	10.02 (0.20%)	9.90 (1.00%)
	R3	10.38 (3.80%)	10.73 (7.30%)	9.35 (6.50%)	10.00 (0.00%)	10.01 (0.10%)	10.00 (0.00%)	9.94 (0.60%)	10.00 (0.00%)	10.34 (3.40%)
	R4	10.38 (3.80%)	10.73 (7.30%)	9.35 (6.50%)	10.00 (0.00%)	10.01 (0.10%)	10.00 (0.00%)	10.18 (1.80%)	10.08 (0.80%)	10.00 (0.00%)
	R5	10.38 (3.80%)	10.73 (7.30%)	9.35 (6.50%)	10.00 (0.00%)	10.01 (0.10%)	10.00 (0.00%)	10.08 (0.80%)	9.78 (2.20%)	10.06 (0.60%)
15	R1	15.54 (3.60%)	16.13 (7.53%)	14.33 (4.47%)	15.00 (0.00%)	15.01 (0.07%)	15.00 (0.00%)	14.56 (2.93%)	14.72 (1.87%)	14.81 (1.27%)
	R2	15.54 (3.60%)	16.13 (7.53%)	14.33 (4.47%)	15.00 (0.00%)	15.01 (0.07%)	15.00 (0.00%)	14.98 (0.13%)	14.94 (0.40%)	14.93 (0.47%)
	R3	15.54 (3.60%)	16.13 (7.53%)	14.33 (4.47%)	15.00 (0.00%)	15.01 (0.07%)	15.00 (0.00%)	15.16 (1.07%)	14.91 (0.60%)	15.16 (1.07%)
	R4	15.54 (3.60%)	16.13 (7.53%)	14.33 (4.47%)	15.00 (0.00%)	15.01 (0.07%)	15.00 (0.00%)	14.91 (0.60%)	14.48 (3.47%)	15.24 (1.60%)
	R5	15.54 (3.60%)	16.13 (7.53%)	14.33 (4.47%)	15.00 (0.00%)	15.01 (0.07%)	15.00 (0.00%)	15.10 (0.67%)	15.13 (0.87%)	14.89 (0.73%)

**Table 6:** Damage extent detection results of three methods under various speeds and roughness.

Designed Damage Extent (%)	Detected Damage Extent (%)								
	M1			M2			M3		
	Noise-Free	50	20	Noise-Free	50	20	Noise-Free	50	20
5	5.18 (3.60%)	5.38 (7.60%)	2.18 (56.40%)	5.00 (0.00%)	5.01 (0.20%)	5.11 (2.20%)	5.02 (0.40%)	5.31 (6.20%)	7.07 (41.40%)
10	10.38 (3.80%)	10.73 (7.30%)	5.21 (47.90%)	10.00 (0.00%)	10.02 (0.20%)	10.27 (2.70%)	10.24 (2.40%)	11.32 (2.91%)	11.73 (6.64%)
15	15.54 (3.60%)	15.98 (6.53%)	11.86 (20.93%)	15.00 (0.00%)	15.01 (0.07%)	15.38 (2.53%)	14.56 (2.93%)	14.92 (0.53%)	15.25 (1.67%)
